# Triple-negative mouse breast cancer initiating cells show high expression of beta1 integrin and increased malignant features

**DOI:** 10.1515/biol-2022-0510

**Published:** 2023-03-03

**Authors:** Jing Fu, Shengkun Peng

**Affiliations:** Department of Breast Surgery, Sichuan Academy of Medical Sciences, Sichuan Provincial People’s Hospital, Chengdu, Sichuan, 610000, China; Department of Radiology, Sichuan Academy of Medical Sciences, Sichuan Provincial People’s Hospital, No. 32 of West Section 1st Ring Road, Chengdu, Sichuan, 610000, China

**Keywords:** β1 integrin, extracellular matrix, focal adhesion kinase, metastasis, triple-negative breast cancer

## Abstract

Triple-negative breast cancer (TNBC) is a subtype of breast cancer that exhibits aggressive tumor phenotypes, including rapid metastasis and tumor recurrence. Integrins belong to the family of transmembrane glycoproteins involved in regulating cell adhesion, proliferation, and differentiation through cell–cell and cell–extracellular matrix interactions. Aberrant β1 integrin signaling has been implicated in cancer invasion and metastasis processes. The present work aimed to investigate the role of β1 integrin in TNBC cancer progression using a mouse 4T1 cell line as a model system. We have sorted a subset of tumor-initiating cells (TICs) from the 4T1 cell line based on CD133 positivity by flow cytometry. RT-PCR and protein analysis studies showed the transcriptional upregulation of β1 integrin and its downstream target focal adhesion kinase in 4T1-TICs compared to parental 4T1 cells. In addition, the expression of β1 receptors in TICs is significantly higher than in parental population cells. Furthermore, *in vitro* cellular assays revealed that CD133^+^ TICs have higher clonogenic ability, invasion, and sphere formation potential. These findings suggest that β1 integrin has a potential role in TNBC invasion and metastasis. Hence, β1 integrin could be a possible factor for future targeted cancer therapies.

## 1 Introduction

Triple-negative breast cancer (TNBC) is fundamentally characterized by the absence of estrogen receptors, progesterone receptors, and human epidermal growth factor receptor 2. Notably, there is no precise and targeted therapeutics available for TNBC and thus resulting in a patient’s poor survival rate. Due to the aggressive proliferation rate, poor prognosis, and rapid metastasis to distant sites (lungs, liver, and brain), TNBC hampers current chemotherapy and causes tumor recurrence [1,2]. Tumor recurrence has been reported to occur within 3 years after the first chemotherapy, with most deaths occurring in the first 5 years [3]. Mutations in the breast cancer 1 gene are the early onset for TNBC, and the subset of TNBC became highly metastatic with extremely poor prognosis and became chemotherapy resistant [4]. The small subset of self-renewing tumor cells that can initiate tumor growth is cancer stem cells (CSCs) or tumor-propagating cells (TICs). Importantly, these cells are highly chemoresistant, tumorigenic, and self-renewal due to the overexpression of cell surface antigens such as CD133, CD44, and CD22 [5]. CD133 is a surface glycoprotein widely used as a surface marker to identify CSC in many human tumors [6]. CD133^+^ cancer cells possess higher tumorigenic activity and are resistant to anticancer drugs and radiation [7]. Such cell population includes a more remarkable ability to form floating spheres and tumor mass than CD133^−^ cells. Furthermore, studies have demonstrated that tumor-initiating cells (TICs) can be isolated based on CD133 positivity and CD133^+^ TICs could promote tumor growth in NOD/SCID mice [8]. Also, it has been shown that a higher percentage of CD44^+^/CD24^+^ expressing cells were found in TNBC compared to other breast cancer subtypes [9]. Hence, understanding the molecular mechanism of TICs-mediated tumorigenesis, metastasis, and invasion is an urgent need for developing targeted therapeutics.

Most cell–cell and cell–extracellular matrix (ECM) interactions are mediated by the integrins, a transmembrane heterodimeric protein containing α and β subunits. Amongst, β1 integrin was found to be abnormally activated in human breast carcinoma, which drives malignant phenotypes such as epithelial-to-mesenchymal transition, metastasis, invasion, angiogenesis, and also affects therapeutic modalities [10,11]. Studies on human breast cancer xenograft and treatment with a blocking agent against β1 integrin showed partial attenuation of tumor growth by inducing apoptosis [12,13]. In contrast, different breast cancer cells observed a significant inhibitory effect [14]. Consequently, understanding the targeting factors and multifunctional pathway of β1 integrin in breast cancer would undoubtedly enhance the treatment efficacy and outcomes.

The various downstream signaling targets of β1 integrin act as driving forces for tumor progression, including PI3K, ERK/MAPK, and focal adhesion kinase (FAK) [15,16]. Among these, the FAK is phosphorylated at the Y397 residue upon activation by integrin, which leads to aggressive cancer features such as enhanced cell spreading, cell proliferation, and invasion through multifaceted FAK-associated signal transduction pathways [17,18]. Indeed, overexpression of FAK has been reported in metastatic breast cancer, and the FAK inhibition indeed reduced the metastatic features. Thus, integrin-mediated FAK activation should be considered as a potential target to impede the aggressive cancer phenotypes of breast cancer. Aberrant activation and crosstalk between β1 integrin and other cellular targets are crucial for breast cancer carcinogenesis [19]. Remarkably, β1 integrin involved coordination and crosstalk with various signals to maintain the wide range of cellular functions. Deregulation of β1 integrin and its downstream signaling cascades favor mammary tumor development and progression [20]. The regulation of β1 integrin in these complex processes remains unclear. In the present study, we have used mouse TNBC cell line 4T1 to study the role of β1 integrin in breast cancer progression. We have sorted tumor-initiating TICs from 4T1 cells based on CD133 expression by flow cytometry and further investigated the potential mechanism of TICs-mediated TNBC metastasis and invasion. Our findings suggest that β1 integrin has a potential role in TNBC invasion and metastasis.

## 2 Materials and methods

### 2.1 Cells and culture conditions

Mouse TNBC cell line 4T1 was purchased from the Chinese Academy of Sciences, Shanghai. Monolayered cells were cultured in Dulbecco’s modified Eagle’s medium (DMEM) provided with 10% fetal bovine serum (FBS) and required antibiotics. Cells were incubated at 37°C with 5% CO_2_ in a humidified atmosphere.

### 2.2 Flow cytometry and cell sorting

In brief, 4T1 cells were seeded overnight onto sterile cultural plates at a 1.5 × 10^3^ cells/plate density. For the TICs isolation, cells were stained with anti-CD133-FITC (Thermo Fisher; 1:200) and unstained cells were used as negative controls. After washing the samples with PBS solution three times, cells were resuspended in 500 μL PBS and then subjected to flow cytometry cell sorting. BD FACSort (BD Biosciences) was used for cell sorting. Live cells were gated as P1 population, and dead cells/cell debris were excluded by increasing the height of the photomultiplier threshold for forward scattering. The flow rate was set as 100 cells/s and the sorted cells were collected in a separate sterile tube containing a culture medium. The sorted cells were maintained at 37°C with 5% CO_2_ in a humidified atmosphere. To measure the mean fluorescence signal intensity, cells were stained with anti-CD29-FITC (Thermo Fisher; 1:100) and incubated on ice for 1 h. Subsequently, cells were washed with 1× PBS three times and subjected to fluorescence activated cell sorting analysis. The values presented in the quantification graphs are the average values of three independent experiments.

### 2.3 RT-PCR analysis

RNA was obtained from cells using the TRIzol method and then reversed to cDNA with RT Kit Superscript III (Invitrogen). Quantitative PCR was carried out using SYBRGreen (Takara) with appropriate primers against β1 integrin and GAPDH designed by Primer 5.0. The reaction conditions include an initial step of 10 min at 95°C and then 40 cycles of amplification, which includes 10 s at 95°C, 20 s at 58°C, and 25 s at 72°C. Quantification was determined by 2^−ΔΔCT^ [21]. The internal control used was GAPDH.

β1 integrin – Forward primer: GAGGTTCAATTTGAAATTAGC and Reverse primer: GGCTCTGCACTGAACACATTC; GAPDH – Forward primer: ACGACCCCTTCATTGACCTC and Reverse primer CTTTCCAGAGGGGCCATCCAC.

### 2.4 Soft agar assay

The assay was performed as per the previously described protocol [22]. Cells were mixed with 0.3% agarose in DMEM and layered on top of 0.5% agar (acts as a base layer for the plate) in DMEM supplemented with 10% FBS and incubated at 37°C. The medium was changed every 2 days, and after 20 days the plates were stained with 0.005% crystal violet. The colonies were visualized under a microscope and the number of colonies were counted for three independent experiments.

### 2.5 Matrigel invasion assay

Cells cultured in DMEM in BD matrigel invasion chambers at 37°C for 48 h were subjected to swabbing matrigel top layer with Q-tip, and therefore, non-invading cells were washed off. The invading cells on the membrane were stained with hematoxylin, mounted on slides and visualized under a microscope at 40× objective lens. The number of invading cells was counted, and the values were represented as a quantitative graph.

### 2.6 Sphere formation assay

We performed the sphere formation assay as per the previously described protocol [23]. The flow cytometry sorted TICs and parental 4T1 cells were grown in a serum-free 1:1 mixture of Ham’s F-12/DMEM supplemented with N_2_ and bFGF (10 ng/mL) and EGF (10 ng/mL) at a density of 10^3^ cells/mL in a 6-well plate. Spheres >50 µm diameter were counted after 2 weeks. Sphere formation efficiency was calculated using the formula: (Total number of spheres formed/total number of live cells seeded) × 100.

### 2.7 Protein separation and western blot analysis

Protein lysate was prepared from the samples using a standard protocol and was separated in 10% SDS gel as described previously [24]. Proteins were transferred (using the wet transfer method) to the PVDF membrane. After transfer, the membrane was blocked using 5% BSA. After the blocking step, the membrane was incubated with primary antibodies like polyclonal rabbit anti-CD29 (1:1,000; Thermo Fisher), monoclonal rabbit anti-CD44 (1:2,000; Invitrogen), rabbit anti-CD133 (1:1,000; Thermo Fisher), and rabbit anti-GAPDH (1:2,000; Sigma). Blots were developed with HRP-conjugated secondary antibodies, and the protein signal was detected by an enhanced chemiluminescence kit (Amersham Pharmacia Biotech).

### 2.8 Statistical analysis

Student’s *t*-test and one-way analysis of variance (ANOVA) were performed for the comparison between the two groups. The results presented in the graphs are mean  ±  SEM (standard error of mean) and the *p*-values such as *p  <  0.05 and **p  <  0.01 are considered statistically significant.

## 3 Results

### 3.1 Overexpression of β1 integrin and FAK in CD133^+^ mouse TNBC 4T1-TICs

**Figure 1:**
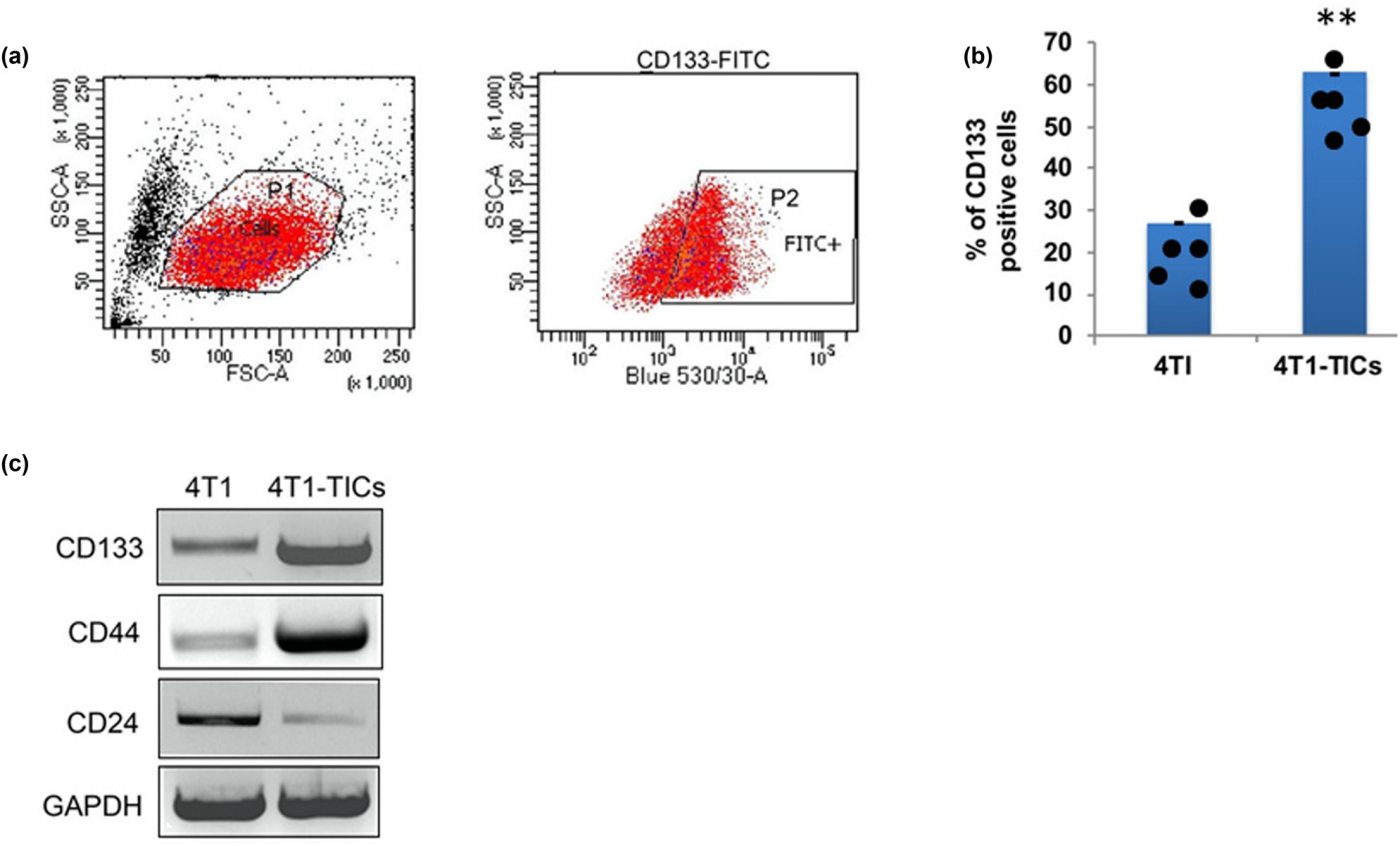
Sorting of CD133^+^ TICs from mouse TNBC 4T1 cell line. (a) Flow cytometry-based sorting of TICs. Live cells selected in the P1 gated region and TICs were isolated based on CD133 fluorescence intensity (P2 gated region). (b) Quantification graph showing percentage of TICs in mouse TNBC 4T1 cells. (c) Western blot showing that isolated 4T1-TICs displayed overexpression of stem cell surface markers like CD133 and CD44, while as downregulation of CD24. The values represented in the quantitative data as means  ±  SEM (*p  <  0.05; **p  <  0.01).

**Figure 2:**
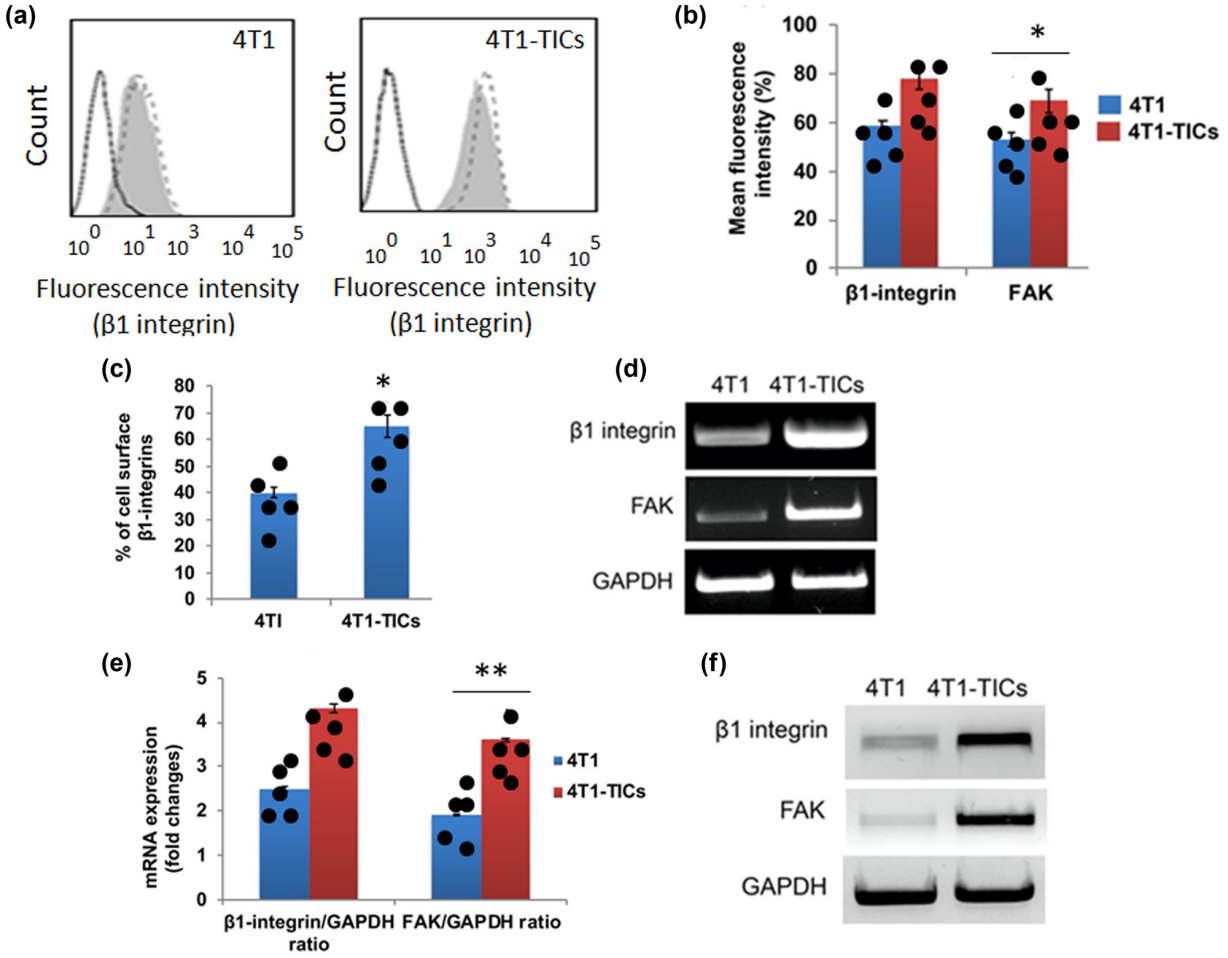
Overexpression of β1 integrin in mouse TNBC 4T1-TICs. Quantification graphs from flow cytometry analysis (a–c) showing significantly higher β1 integrin signal and cell surface receptors in sorted TICs, respectively. RT-PCR (d and e) and western blot data (f) significantly enhanced transcriptional upregulation and protein overexpression in 4T1-TICs. The values represented in the quantitative data as means  ±  SEM (*p  <  0.05; **p  <  0.01).

To determine the factors and signaling pathways involved in breast cancer metastasis and invasion, we have used breast mouse adenocarcinoma cell line 4T1 as a model system in this study. TICs possess the characteristic features of stem cells, such as high proliferation, self-renewal, and enhanced stem cell surface markers [25]. For isolating a population of TICs, cells were stained with CD133-FITC antibody to differentiate and sort the population of cancer stem-like TICs. By flow cytometry, we have isolated a sub-population of TICs whose mean intensity for CD133-FITC signal (P2 gated region) is significantly higher than parental 4T1 cells (Figure 1a) and accounts for more than 25% of the total cell population (Figure 1b). Furthermore, protein analysis confirmed that sorted 4T1-TICs displayed higher expression of CD133 and cell surface glycoproteins such as CD24 and CD44 [26], which are essential for cell adhesion and migration (Figure 1c). Next, we analyzed and compared the expression profile of β1 integrin between sorted TICs and parental mouse 4T1 cells. Flow cytometry was performed to measure and quantify the mean intensity of β1 integrin (CD29-FITC). As a result, we found that β1 integrin signaling is significantly overexpressed in sorted TICs (Figure 2a). Consequently, the cell surface level of β1 integrin is significantly higher in 4T1-TICs, rather than parental 4T1 cells (Figure 2b). In addition, RT-PCR and protein analysis data revealed that TICs has enhanced transcriptional regulation (*p* < 0.01), and protein expression was observed for the β1 integrin gene (Figure 2c–e). Similarly, the FAK, which is crucial for cell migration and invasion by interaction with integrins, is also upregulated in TICs (Figure 2c–e). Therefore, these results suggest that β1 integrin is overexpressed in mouse breast adenocarcinoma TICs isolated from the 4T1 cell line. Furthermore, these cells also possess enhanced expression of protein, which are crucial for cancer cell adhesion, migration, and invasion.

### 3.2 CD133^+^ mouse TNBC 4T1-TICs display more aggressive tumorigenic features compared to 4T1 cells

**Figure 3:**
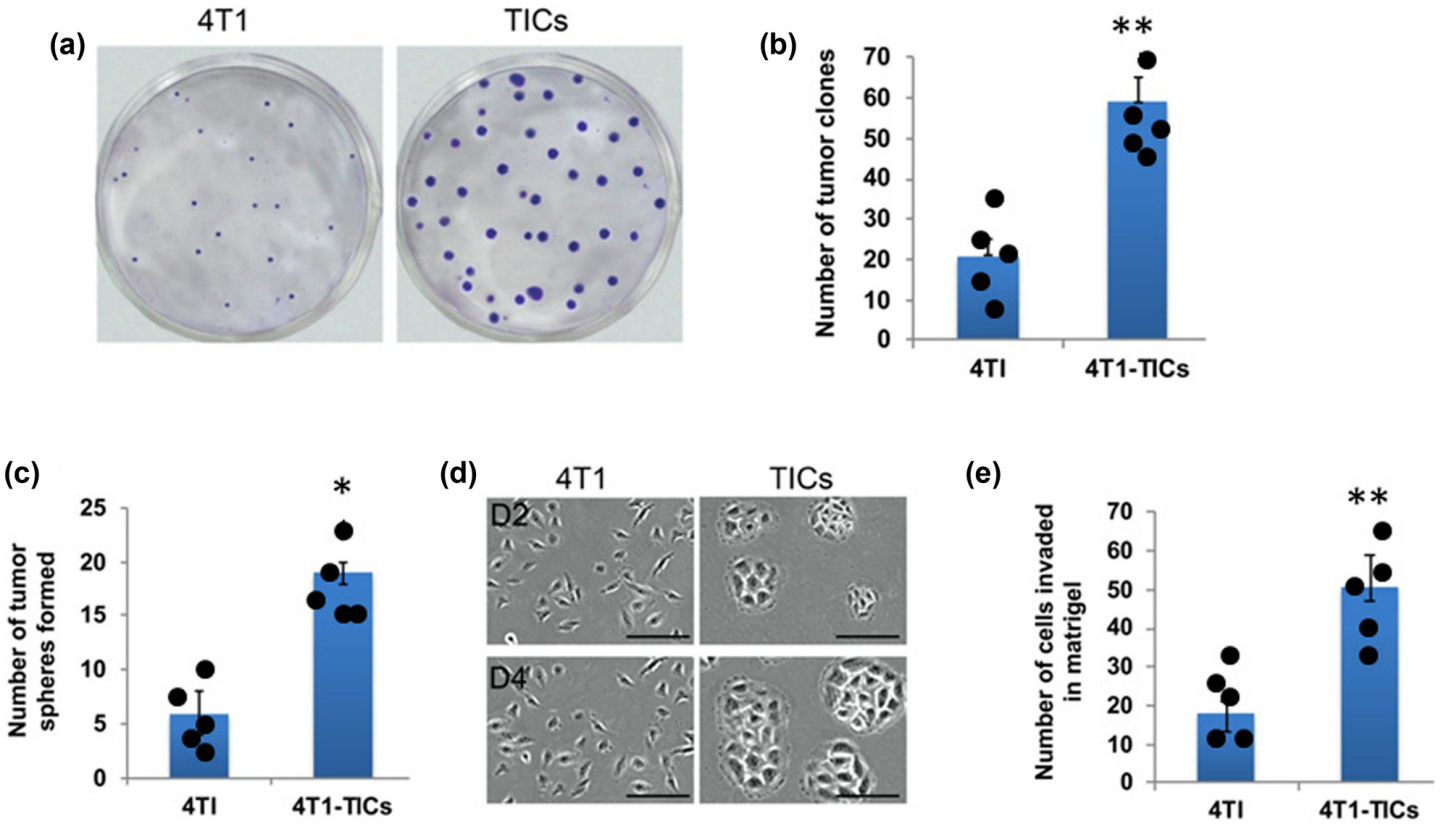
Effect of β1 integrin overexpression in mouse TNBC malignancy. Soft agar assay (a and b) showing that 4T1-TICs able to produce bigger and greater number of tumor clones rapidly. Similarly, the TICs were able to generate tumorspheres efficiently and the size increases over the time (c and d). (e) TICs were able to invade through the matrigel more efficiently than parental 4T1 cells. The values represented in the quantitative data as means  ±  SEM (*p  <  0.05; **p  <  0.01).

The flow cytometer sorted TICs displayed overexpressed stem cell surface glycoproteins essential for self-renewal, cell adhesion, migration, and invasion [5]. Hence, we compared the clonogenic ability, invasion, and tumorsphere-forming potential between TICs and parental 4T1 cells. The TICs can produce more tumor clones on soft agar plates than 4T1 cells (Figure 3a and b). Furthermore, the TIC-produced tumor clones grew more prominent and faster, whereas the clones produced by 4T1 were relatively smaller in size and had slower growth. Similarly, the sphere formation assay showed that TICs could produce more tumorspheres expeditiously, and their size increases with time (Figure 3c and d). Subsequent matrigel invasion assay confirmed the increased migratory potential of TICs. The number of cells migrated/invaded through the matrigel is significantly higher in TICs compared to 4T1 cells (Figure 3e). Altogether, these findings suggest that sorted CD133^+^ TICs from mouse 4T1 cell line possess strong clonogenic and higher tumor invasion properties, possibly owing to overexpression of β1 integrin and FAK.

## 4 Discussion

Integrins are essential for cell motility, proliferation, and survival. Increasing evidence shows the implications of dysregulated integrin signaling in breast cancer tumorigenesis and progression [27]. In the present study, we found higher β1 integrin and FAK expression in CD133^+^ TIC from highly metastatic and invasive mouse TNBC cell line 4T1. A subset of a small population of TICs was isolated by flow cytometry based on increased positive staining of stem cell surface protein CD133. It has already been reported that overexpression of stemness proteins contributes to high self-renewal, tumorigenic, and differentiation potential of CSCs or TICs [8,28]. These TICs showed an increased mRNA and protein expression of β1 integrin compared to parental 4T1 cells. Previous reports in human TNBC cell lines also showed aberrant regulation of integrins and the role of distinct integrins in breast cancer metastasis [14]. Abundantly expressed β1 integrin was associated with high metastasis with non-small-cell lung carcinoma [29]. In contrast, downregulated β1 integrin expression promotes carcinogenesis of pancreatic cancer [30]. However, the underlying mechanism of integrin-mediated breast cancer progression remains unclear. Hence, it is a pressing quest to unravel the factors and mechanisms involved in the β1 integrin-mediated tumorigenesis to solve these discrepancies.

β1 integrins can stimulate and activate downstream targets such PI3K, ERK/MAPK, EGFR, and FAK, which drive cell–ECM interaction for cell proliferation events [14]. At the same time, β1 integrin knockdown and treatment with a blocking agent against β1 integrin can suppress the aggressive phenotypes of breast cancer by inducing apoptotic cell death [12,13]. Our study showed that FAK is also overexpressed as β1 integrin in CD133^+^ TNBC-TICs. It is well documented that FAK expression is correlated with breast malignancy progression and poor clinical outcomes. Hence, FAK overexpression is associated with aggressiveness in breast tumorigenesis, metastasis, and invasion [31,32]. Consistent with the previous report, β1 integrin and FAK overexpressing TNBC 4T1-TICs have high clone formation efficiency, invading through matrigel and tumorsphere formation. This finding confirms the tumorigenic and invasion role of TICs. FAK activation might involve integrin–ECM interaction and further clustering of proteins (such as paxillin) at focal adhesion sites with integrins [33]. Another possible underlying mechanism of FAK-mediated tumorigenesis might be the activation of FAK downstream cascades such as Src and PI3/AKT signaling pathways, which are crucial for unlimited cell proliferation by impeding apoptosis [34]. Nonetheless, reports suggest that FAK is stimulated by various extracellular factors such as cytokines, lipid mediators, growth factors, and GPCRs pathways [31]. In this respect, studies have used FAK inhibitors and observed promising outcomes such as the prevention of cancer cell migration, particularly in TNBC cells [31].

In summary, our results showed that β1 integrin and FAK are overexpressed in TICs of mouse TNBC 4T1 cells. Notably, these findings are the outcome of fundamental research. Furthermore, knockdown approaches and animal models would shed more light on the molecular mechanism of β1 integrin mediating tumorigenesis in TICs of breast cancer. Meanwhile, considering the carcinogenic role of deregulated β1 integrin and FAK, new anti-cancer drugs should be designed to target them to enhance the treatment efficacy.
